# 2279 Understanding care delivered to patients with a possible concussion at an urban level 1 trauma center

**DOI:** 10.1017/cts.2018.309

**Published:** 2018-11-21

**Authors:** Chet C. Zalesky, David W. Wright, Sanam Patel, Rachel K. Patzer

**Affiliations:** 1 Emory University; 2 Department of Emergency Medicine, Injury Prevention Research Center, Emory University School of Medicine, Emory University; 3 Medical College of Georgia, AU/UGA Medical Partnership; 4 Department of Emergency Medicine, Emory University School of Medicine

## Abstract

OBJECTIVES/SPECIFIC AIMS: Background: Annually, 2.5 million traumatic brain injuries (TBI) occur with nearly 75% classified as mild TBI (mTBI), also known as a concussion. Mild TBI can be subtle and detection requires a high index of suspicion and a regimented evaluation process. This study was done to define the proportion of patients with a possible mTBI evaluated for concussion at a high volume urban trauma center. METHODS/STUDY POPULATION: Methods: A prospective cohort of patients was identified using a 3-question screen at the time of triage: did an injury occur; was the mechanism consistent with mTBI; was there a period of altered mental status. Patients who screened positive were thought to meet a minimum threshold for the evaluation of mTBI. Information about mTBI specific evaluation, management, and education was obtained from the patient’s charts. RESULTS/ANTICIPATED RESULTS: Results: 38,484 patients were screened over 16 weeks, of whom 453 (1.18%) screened positive for a possible mTBI and did not meet exclusion criteria. In total, 198 patients had documented loss of consciousness, 101 were diagnosed with mTBI, and 49 received mTBI discharge instructions. Overall, 32.5% of included patients had mTBI listed in the differential or as a diagnosis and 32.3% with loss of consciousness received a mTBI diagnosis. DISCUSSION/SIGNIFICANCE OF IMPACT: Conclusions: Many patients with a possible mTBI were not evaluated, managed, or educated for their potential injury. Changes in physicians’ approach to mTBI must occur to increase the proportion of patients receiving appropriate evaluation, management, and education. These results define the current reality of mTBI treatment in the Emergency Department and show the need for further experimental studies targeted at physician decision support interventions to improve mTBI care.

